# L-shaped association of serum calcium with all-cause and CVD mortality in the US adults: A population-based prospective cohort study

**DOI:** 10.3389/fnut.2022.1097488

**Published:** 2023-01-05

**Authors:** Xinran Hou, Jie Hu, Zhuoyi Liu, E. Wang, Qulian Guo, Zhong Zhang, Zongbin Song

**Affiliations:** ^1^Department of Anesthesiology, Xiangya Hospital, Central South University, Changsha, China; ^2^National Clinical Research Center for Geriatric Disorders, Xiangya Hospital, Central South University, Changsha, China

**Keywords:** serum calcium, mortality, all-cause, cardiovascular disease, National Health and Nutrition Examination Survey (NHANES)

## Abstract

**Background:**

Calcium is involved in many biological processes, but the impact of serum calcium levels on long-term mortality in general populations has been rarely investigated.

**Methods:**

This prospective cohort study analyzed data from the National Health and Nutrition Examination Survey (1999–2018). All-cause mortality, cardiovascular disease (CVD) mortality, and cancer mortality were obtained through linkage to the National Death Index. Survey-weighted multivariate Cox regression was performed to compute hazard ratios (HRs) and 95% confidential intervals (CIs) for the associations of calcium levels with risks of mortality. Restricted cubic spline analyses were performed to examine the non-linear association of calcium levels with all-cause and disease-specific mortality.

**Results:**

A total of 51,042 individuals were included in the current study. During an average of 9.7 years of follow-up, 7,592 all-cause deaths were identified, including 2,391 CVD deaths and 1,641 cancer deaths. Compared with participants in the first quartile (Q1) of serum calcium level [≤2.299 mmol/L], the risk of all-cause mortality was lower for participants in the second quartile (Q2) [2.300–2.349 mmol/L], the third quartile (Q3) [2.350–2.424 mmol/L] and the fourth quartile (Q4) [≥2.425 mmol/L] with multivariable-adjusted HRs of 0.81 (95% CI, 0.74–0.88), 0.78 (95% CI, 0.71–0.86), and 0.80 (95% CI, 0.73, 0.88). Similar associations were observed for CVD mortality, with HRs of 0.82 (95% CI, 0.71–0.95), 0.87 (95% CI, 0.74–1.02), and 0.83 (95% CI, 0.72, 0.97) in Q2–Q4 quartile. Furthermore, the L-shaped non-linear associations were detected for serum calcium with the risk of all-cause mortality. Below the median of 2.350 mmol/L, per 0.1 mmol/L higher serum calcium was associated with a 24% lower risk of all-cause mortality (HR: 0.76, 95% CI, 0.70–0.83), however, no significant changes were observed when serum calcium was above the median. Similar L-shaped associations were detected for serum calcium with the risk of CVD mortality with a 25% reduction in the risk of CVD death per 0.1 mmol/L higher serum calcium below the median (HR: 0.75, 95% CI, 0.65–0.86).

**Conclusion:**

L-shaped associations of serum calcium with all-cause and CVD mortality were observed in US adults, and hypocalcemia was associated with a higher risk of all-cause mortality and CVD mortality.

## Introduction

Calcium is vital to many biological processes, besides its most widely recognized role as an essential structural component of the skeleton (1). The serum calcium, representing the extracellular calcium, could act on calcium-sensing receptors on many types of cells, modulate the excitability of nervous tissues and muscle tissues by impacts on cell membrane potentials, and function as a co-factor for various enzymes, such as those involved in blood coagulation (2). The serum calcium homeostasis is tightly regulated by parathyroid hormone, calcitonin, and 1,25-dihydroxyvitamin D; and three forms of serum calcium, ionized (51%), complexed (9%), and protein-bound (40%), keep in stable and dynamic equilibrium (3, 4).

Recently, it has been realized that the altered serum calcium hemostasis may have more clinical significance, than just indicating the dysfunction of calcium metabolism. The association of serum calcium with mortality has been investigated in certain populations, such as patients with chronic heart failure (5), coronary heart disease (6), hemodialysis (7–9), and ischemic stroke (10). However, the evidence for the association of serum with mortality from the general population is rare and limited.

In the present study, we aimed to investigate the association of serum calcium level with all-cause and disease-specific mortality and evaluate the effect size of serum calcium at different levels based on a nationally representative cohort from the National Health and Nutrition Examination Survey (NHANES) 1999–2018 dataset with linkage to the National Death Index (NDI) mortality files.

## Materials and methods

### Study design and population

The NHANES is conducted by the National Center for Health Statistics (NCHS) of the Centers for Disease Control and Prevention, and has been a continuous stratified, multistage survey to assess the health and nutritional status of the resident, civilian, and non-institutionalized population in the U.S. since 1999. Details about NHANES sample design, estimation procedures, and analytic guidelines have been described on the NHANES website (11). NHANES is approved by NCHS Ethics Review Board and written informed consent was obtained from all participants. The data in the present study were publicly released and were used in compliance with its data usage guidelines.

All continuous NHANES (1999–2018) datasets with serum calcium and mortality data were included in the present study. After excluding individuals ages less than 18, during pregnancy, and missing data, the remaining participants were included in the final analysis.

### Measurement of total calcium in serum

Blood samples were collected and processed in the mobile examination center by certified laboratory professionals, then stored in biorepositories. Total calcium was determined as part of the routine biochemistry profile with Synchron LX20 (Beckman Coulter, Inc.) or UniCel^®^ DxC 800 Synchron (Beckman Coulter, Inc.), both of which used indirect (or diluted) ion selective electrode (I. S. E. Wang.) methodology to measure calcium concentration in serum. The calcium in mg/dL was converted to mmol/L by multiplying by 0.250. Details about the laboratory procedure have been described on the NHANES website (12).

### Ascertainment of all-cause mortality and disease-specific mortality

All-cause and disease-specific mortality were obtained through linkage to the NDI (13), in which the public-use Linked Mortality Files (LMF) are available for 1999–2018 NHANES. All-cause mortality was defined as death from any cause; cardiovascular disease (CVD) mortality was defined as codes I00–I09, I11, I13, I20–I51, and I60–I69 (Supplementary Table 1); cancer mortality was defined as codes C00–C97 (Supplementary Table 2) according to the International Classification of Diseases, 10th Revision (ICD-10) codes recorded as the underlying cause of death.

The follow-up time referred to the months from the date of the NHANES examination to the date of death or the end of the follow-up period (31 December 2019) for those who were censored.

### Assessment of other covariates

The baseline demographic characteristics, lifestyle factors, and medical conditions collected during the in-person household interviews were selected based on previous literature to reduce the effect of potential confounding (14–16).

Among the demographic characteristics, the race/Hispanic origin categories were as follows: non-Hispanic white (white), non-Hispanic black (black), Mexican American, and others. Education level was categorized into less than 9th grade, 9–11th grade, high school, college or AA degree, and college graduate or above. Marital status was classified into married, widowed, divorced, separated, never married, and living with a partner. Income level was measured based on the family poverty-to-income ratio (PIR), which is a ratio of family income to the poverty threshold, continuous from 0 to 5, and a higher value indicates higher family income per capita.

Among the lifestyle factors, body mass index (BMI) was calculated as weight in kilograms divided by the square of height in meters. Smoking status was classified into never, former, and current smoker, based on responses to the following two questions: “Have you smoked at least 100 cigarettes in your entire life?” and “Do you now still smoke cigarettes?” Alcohol user was defined as never (had <12 drinks in lifetime), former (had ≥ 12 drinks in lifetime but did not drink last year), mild (≤1 drink per day for women or ≤2 drinks per day for men), moderate (≤2 drink per day for women or ≤3 drinks per day for men), and heavy (>2 drink per day for women or >3 drinks per day for men). Caffeine consumption was evaluated as the mean consumption from 2 typical days 3–10 days intervals and calculated into mg/d (17). Diet quality was assessed using the Healthy Eating Index-2015 (HEI-2015), which ranges from 0 to 100, and a higher score indicates better diet quality (18). The dietary calcium intake (mg) was calculated with nutritional information from foods and beverages collected in two 24-h dietary recalls with 3–10 days intervals. Physical activity was measured NHANES Physical Activity Questionnaire and quantified in metabolic equivalent tasks (METs) minutes per week (METs-min/week) (19).

For comorbid conditions or past medical history, hypertension was determined by (1) SBP ≥ 140 mmHg, (2) DBP ≥ 90 mmHg, (3) have been told by a doctor to have hypertension, or (4) currently taking antihypertensive drug, diabetes; diabetes was determined by (1) fasting glucose ≥ 7.0 mmol/L, (2) glycohemoglobin HbA1c ≥ 6.5%, (3) random blood glucose ≥ 11.1 mmol/L, (4) 2-h OGTT blood glucose ≥ 11.1 mmol/L, (5) have been told by a doctor to have diabetes, or (5) currently use of diabetes medication or insulin; chronic obstructive pulmonary disease (COPD) was determined by (1) FEV1/FVC < 0.7 after use of bronchodilator in spirometry, (2) ever told by a doctor to have COPD, emphysema, or chronic bronchitis, or (3) currently use drugs for COPD; chronic kidney disease (CKD) was determined by eGFR ≤60 ml/min/1.73 m^2^ or albuminuria ≥30 mg/g according to KDIGO recommendation (20); coronary heart disease (CHD), stroke, and cancer were obtained through questionnaires, based on whether participants were ever told by a doctor or other health professionals that they had these conditions.

### Statistical analyses

Statistical analyses were performed according to NHANES recommended guidelines (21, 22). For the complex, multistage probability sampling design in NHANES, examination weights were used to account for non-response and oversampling in all analyses unless otherwise noted. The missing data on covariates were imputed by the multiple imputation method.

Data are presented as survey-weighted mean (standard error, SE) for continuous variables or number (survey-weighted percentage,%) for categorical variables, respectively. Survey-weighted ANOVA and Chi-square test were used to detect statistical differences between the means and proportions between groups.

Multivariate Cox regression models were applied to evaluate the independent association between serum calcium and mortality. Before the inclusion of covariates, collinearity diagnostics were performed to ensure variance inflation factors of all variables less than 5; then they were included when they showed regression coefficients on the outcome with *P*-value less than 0.1 or the matched regression coefficient changed by more than 10% when they were added or removed. The crude and adjusted hazard ratios (HRs) and 95% confidence intervals (CIs) were calculated for the associations of serum calcium to all-cause or disease-specific mortality.

According to the recommendation of Strengthening the Reporting of Observational Studies in Epidemiology (STROBE) statement (23), we simultaneously showed the results of Model 1 (unadjusted), Model 2 (minimally adjusted for sex, age, and race), and Model 3 (additionally adjusted for education, marital status, PIR, BMI, smoke, alcohol use, caffeine consumption, dietary calcium intake, HEI-2015, physical activity, comorbidity or history of hypertension, diabetes, CHD, stroke, COPD, cancer, and CKD).

Survey-weighted multivariate Cox regression with restricted cubic spline analyses was performed to examine the non-linear association of serum calcium levels with all-cause and disease-specific mortality. Models with different numbers of knots were assessed by the Akaike information criterion (AIC); models with smaller AIC values were preferred, in which the minimal knots were chosen with close AIC. Non-linearity was tested using the likelihood ratio test comparing the model with only a linear term against the model with linear and cubic spline terms. Additionally, a segmented weighted Cox proportional hazards model on both sides of the reference value (median of serum calcium) was performed to calculate HRs per 0.1 mmol/L increase in serum calcium after the detection of a non-linear relationship.

The subgroup analyses were performed to demonstrate the associations of serum calcium level in different subgroups stratified by dichotomic variables, namely sex (female or male), age (≤60 or >60), race (white or non-white), education level (high school or below; college or above), marital status (married or other), PIR (<1.3 or ≥1.3), BMI (≤ 28 or >28), smoker (smoker or non-smoker), drinker (current drinker or not), caffeine consumption (less than the median or more than the median), dietary calcium intake (<800 mg or ≥800 mg) HEI-2015 (<50 or ≥50), physical activity (active, namely ≥600 MET-min/week, or inactive, namely <600 MET-min/week), hypertension (yes or no), diabetes (yes or no), stroke (yes or no), COPD (yes or no), cancer (yes or no), and CKD (yes or no), using the fully adjusted model except for the specific stratification variable. Interactions of serum calcium level with stratification variables were also inspected by the likelihood ratio test.

Sensitivity analyses were performed to test the robustness of the association of serum calcium level with mortality. Firstly, participants with serum calcium outside of the mean ± 3*standard deviation were excluded to test the potential effect of extreme value. Secondly, participants who died within 2 years of follow-up were excluded to avoid the potential reverse causation bias. Thirdly, participants with missing data on covariates were excluded to assess the influence of missing variables.

Data were analyzed with the use of the statistical packages in the R program (The R Foundation; version 4.2.0)^1^ and EmpowerStats (X&Y Solutions, Inc., Boston, Massachusetts; version 4.1).^2^ A two-sided *P* < 0.05 was considered significantly different.

## Results

### Selection of participants

**FIGURE 1:**
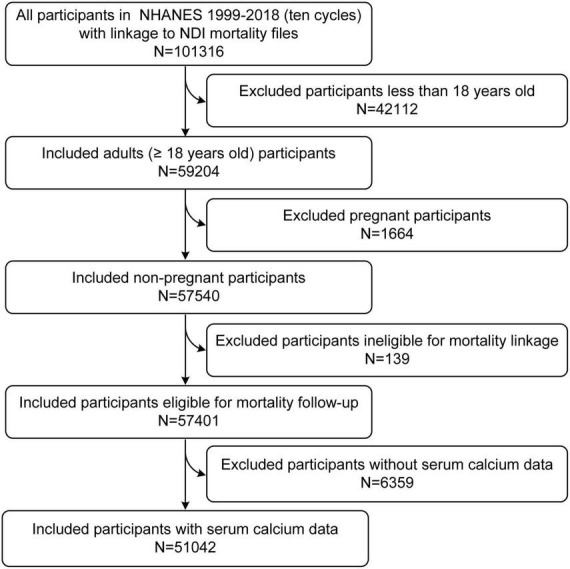
Flowchart of participant enrollment.

The total number of participants in 10 cycles of NHANES (1999–2018) was 101,316. After excluding 42,112 participants with ages less than 18 years old, another 1,664 pregnant participants, another 139 participants ineligible for mortality follow-up (insufficient identifying data), and another 6,359 participants without serum calcium data, 51,042 participants were finally included in our study (Figure 1).

### Baseline characteristics of participants

Of the 51,042 participants which could represent 208 million US adults, 25,621 were female with a weighted percentage of 50.9%; the weighted mean age was 46.3 years; 22,192 were non-Hispanic white with a weighted percentage of 68.5%. The median of serum calcium in the included participants was 2.35 mmol/L [interquartile range (IQR), 2.300–2.425]. During an average of 116.2 months (9.7 years) follow-up, 7,592 all-cause deaths were identified, including 2,391 CVD deaths (weighted percentage, 29.7%) and 1,641 cancer deaths (weighted percentage, 22.9%).

The baseline characteristics of participants by quartile of serum calcium were displayed in Table 1. Briefly, the gender distribution, age, race distribution, education level, marital status, PIR, BMI, smoking, alcohol use, physical activity, dietary calcium intake, HEI-2015, and comorbidity of hypertension, diabetes, stroke, and CKD showed significant differences between Q1-Q4 quartiles of serum calcium (Table 1).

**TABLE 1:** Baseline characteristics of participants in different quartiles of serum calcium.

Variable	Total	Q1 [≤2.299]	Q2 [2.300, 2.349]	Q3 [2.350, 2.424]	Q4 [≥2.425]	*P*-value
Gender (%)						<0.001
Female	25,621 (50.9)	6,299 (58.5)	5,510 (55.0)	7,875 (48.1)	5,937 (45.1)	
Male	25,421 (49.1)	4,850 (41.5)	4,801 (45.0)	8,560 (51.9)	7,210 (54.9)	
Age (years)	46.3 (0.2)	47.9 (0.3)	46.7 (0.2)	45.6 (0.2)	45.4 (0.3)	<0.001
Race (%)						<0.001
White	22,192 (68.5)	4,363 (64.8)	4,404 (68.1)	7,262 (69.2)	6,163 (70.9)	
Black	10,610 (10.7)	2,053 (10.3)	1,996 (10.2)	3,415 (10.6)	3,146 (11.7)	
Mexican	9,388 (8.3)	2,539 (10.8)	2,005 (8.8)	2,935 (7.9)	1,909 (6.3)	
Other	8,852 (12.5)	2,194 (14.2)	1,906 (12.9)	2,823 (12.4)	1,929 (11.1)	
Education (%)						<0.001
Less than 9th grade	5,831 (5.8)	1,560 (6.9)	1,265 (6.1)	1,764 (5.7)	1,242 (5.4)	
9–11th grade	7,032 (10.9)	1,622 (11.4)	1,396 (10.8)	2,225 (11.1)	1,789 (11.8)	
High school graduate/GED	10,987 (23.2)	2,374 (23.6)	2,220 (23.2)	3,462 (23.6)	2,931 (25.6)	
Some college or AA degree	6,166 (13.4)	1,675 (17.1)	1,302 (14.6)	1,986 (13.6)	1,203 (10.8)	
College graduate or above	17,457 (43.3)	3,580 (40.9)	3,607 (45.2)	5,757 (46.0)	4,513 (46.4)	
Marital status (%)						<0.001
Never married	9,901 (18.1)	1,689 (15.0)	1,831 (17.2)	3,340 (19.3)	3,041 (21.9)	
Living with partner	3,529 (7.2)	806 (7.6)	688 (7.0)	1,178 (7.7)	857 (7.3)	
Married	24,912 (53.8)	5,871 (57.8)	5,268 (57.1)	7,958 (55.9)	5,815 (52.0)	
Separated	1,592 (2.4)	393 (2.7)	363 (2.7)	504 (2.5)	332 (2.1)	
Divorced	4,936 (9.6)	1,130 (10.8)	1,004 (10.3)	1,536 (9.3)	1,266 (9.9)	
Widowed	4,153 (5.8)	964 (6.2)	767 (5.7)	1,218 (5.3)	1,204 (6.8)	
PIR	3.0 (0.0)	2.9 (0.0)	3.0 (0.0)	3.0 (0.0)	2.9 (0.0)	<0.001
BMI (kg/m^2^)	28.6 (0.1)	30.1 (0.1)	29.1 (0.1)	28.4 (0.1)	27.5 (0.1)	<0.001
Smoker (%)						<0.001
Never	26,256 (52.6)	5,945 (54.1)	5,551 (55.2)	8,412 (53.9)	6,348 (52.5)	
Former	11,901 (24)	2,846 (26.0)	2,373 (23.8)	3,750 (24.4)	2,932 (24.5)	
Now	10,108 (21)	2,118 (19.9)	2,004 (21.0)	3,314 (21.7)	2,672 (23.0)	
Alcohol user (%)						<0.001
Never	6,533 (10.5)	1,502 (12.6)	1,362 (12.1)	1,974 (10.7)	1,695 (11.8)	
Former	7,601 (12.8)	1,687 (14.0)	1,528 (14.4)	2,394 (14.0)	1,992 (14.8)	
Mild	14,431 (31.9)	3,169 (35.7)	2,952 (35.0)	4,737 (36.3)	3,573 (35.1)	
Moderate	6,461 (15.2)	1,454 (17.5)	1,370 (18.2)	2,112 (16.9)	1,525 (15.8)	
Heavy	8,736 (19.2)	1,845 (20.2)	1,732 (20.4)	2,913 (22.1)	2,246 (22.6)	
Caffeine consumption (mg/day)	176.4 (2.2)	171.9 (3.5)	177.8 (3.6)	179.2 (2.9)	175.3 (3.5)	0.279
Physical activity (METs-min/week)	3,427.1 (61.5)	3,803.2 (120.2)	3,541.8 (97.6)	3,449.4 (84.5)	3,015.9 (91.7)	<0.001
Dietary calcium intake (mg/day)	934.6 (4.9)	897.0 (6.9)	929.7 (8.8)	944.0 (7.3)	956.8 (8.0)	<0.001
HEI-2015 Hypertension (%)	50.2 (0.2)	49.6 (0.3)	50.2 (0.2)	50.4 (0.2)	50.3 (0.2)	0.027 <0.001
No	30,064 (63.2)	6,551 (63.5)	6,188 (65.2)	9,848 (64.2)	7,477 (60.1)	
Yes	20,978 (36.8)	4,598 (36.5)	4,123 (34.8)	6,587 (35.8)	5,670 (39.9)	
Diabetes (%)						0.021
No	42,516 (87.4)	9,123 (86.4)	8,635 (87.5)	13,839 (88.1)	10,919 (87.2)	
Yes	8,522 (12.6)	2,025 (13.6)	1,676 (12.5)	2,595 (11.9)	2,226 (12.8)	
CHD (%)						0.274
No	45,284 (93.1)	10,262 (96.2)	9,332 (96.4)	14,573 (96.8)	11,117 (96.4)	
Yes	2,043 (3.4)	513 (3.8)	427 (3.6)	577 (3.2)	526 (3.6)	
Stroke (%)						<0.001
No	45,645 (93.9)	10,304 (96.5)	9,452 (97.5)	14,700 (97.5)	11,189 (97.2)	
Yes	1,837 (2.7)	510 (3.5)	337 (2.5)	500 (2.5)	490 (2.8)	
COPD (%)						0.247
No	45,561 (92.9)	10,347 (95.6)	9,391 (96.0)	14,591 (96.2)	11,232 (96.2)	
Yes	2,011 (3.8)	486 (4.4)	416 (4.0)	633 (3.8)	476 (3.8)	
Cancer (%)						0.163
No	43,089 (87.6)	9,782 (89.8)	8,927 (90.7)	13,816 (90.9)	10,564 (90.9)	
Yes	4,404 (9.1)	1,040 (10.2)	865 (9.3)	1,382 (9.1)	1,117 (9.1)	
CKD (%)						<0.001
No	41,277 (84.9)	8,854 (85.5)	8,425 (85.9)	13,535 (86.7)	10,463 (84.5)	
Yes	9,217 (14.1)	2,134 (14.5)	1,793 (14.1)	2,731 (13.3)	2,559 (15.5)	

Continuous variables are presented as survey-weighted mean (SE) and categorical variables are presented as numbers (survey-weighted percentage, %).

CI, confidence interval; BMI, body mass index; PIR, family income-to-poverty ratio; GED, general educational development; AA, associate of arts; HEI, healthy eating index; COPD, chronic obstructive pulmonary disease; CHD, coronary heart disease; CKD, chronic kidney disease.

### Multivariate cox regression analysis of the association between serum calcium quartiles with mortality

In all three models adjusted for different covariates, the all-cause mortality in the second, third, and fourth quartile of serum calcium (Q2–Q4) was consistently lower than that in the reference group (Q1). Specifically, after full adjustment, compared with the first quartile, the HRs for all-cause mortality were 0.81 (95% CI, 0.74–0.88) in the second quartile, 0.78 (95% CI, 0.71–0.86) in the third quartile, and 0.80 (95% CI, 0.73–0.88) in the fourth quartile (Table 2).

**TABLE 2:** Multivariate analysis of the association between serum calcium level quartiles and mortality.

	Q1 [≤2.299]	Q2 [2.300, 2.349]		Q3 [2.350, 2.424]		Q4 [≥2.425]	
	HR (95% CI)	HR (95% CI)	*P*	HR (95% CI)	*P*	HR (95% CI)	*P*
**All-cause mortality**							
Model 1	1 (ref)	0.78 (0.71, 0.85)	<0.001	0.77 (0.70, 0.84)	<0.001	0.82 (0.75, 0.90)	<0.001
Model 2	1 (ref)	0.80 (0.74, 0.88)	<0.001	0.81 (0.74, 0.88)	<0.001	0.84 (0.78, 0.92)	<0.001
Model 3	1 (ref)	0.81 (0.74, 0.88)	<0.001	0.78 (0.71, 0.86)	<0.001	0.80 (0.73, 0.88)	<0.001
**CVD mortality**							
Model 1	1 (ref)	0.78 (0.67, 0.91)	0.002	0.83 (0.71, 0.97)	0.018	0.85 (0.73, 0.98)	0.024
Model 2	1 (ref)	0.82 (0.70, 0.95)	0.008	0.88 (0.75, 1.03)	0.120	0.87 (0.75, 1.01)	0.061
Model 3	1 (ref)	0.82 (0.71, 0.95)	0.010	0.87 (0.74, 1.02)	0.094	0.83 (0.72, 0.97)	0.021
**Cancer mortality**							
Model 1	1 (ref)	0.82 (0.69, 0.98)	0.029	0.79 (0.66, 0.94)	0.009	0.79 (0.68, 0.93)	0.004
Model 2	1 (ref)	0.84 (0.70, 1.02)	0.077	0.83 (0.69, 0.99)	0.034	0.82 (0.69, 0.97)	0.024
Model 3	1 (ref)	0.84 (0.70, 1.02)	0.087	0.81 (0.68, 0.97)	0.022	0.80 (0.68, 0.95)	0.009

Data were calculated by svycoxph to fit a multivariate Cox’s proportional hazards model to data from a complex survey design.

Model 1: Unadjusted.

Model 2: Adjusted for sex, age, and race.

Model 3: Adjusted for sex, age, race, education, marital status, family income-to-poverty ratio, BMI, smoking, alcohol use, caffeine consumption, dietary calcium intake, HEI-2015, physical activity, comorbidity or history of hypertension, diabetes, CHD, stroke, COPD, cancer, and CKD.

HR, hazard ratio; CI, confidential interval; CVD, cardiovascular disease.

In all three models, the CVD mortality in the second quartile (Q2) tended to be lower than that in the reference group (Q1). Specifically, after full adjustment, compared with the first quartile, the HRs for CVD mortality were 0.82 (95% CI, 0.71–0.95) in the second quartile, 0.87 (95% CI, 0.74–1.02) in the third quartile, and 0.83 (95% CI, 0.72–0.97) in the fourth quartile (Table 2).

In the crude and fully adjusted models, the cancer mortality in the third and fourth quartile (Q3–Q4) tended to be lower than that in the reference group (Q1). Specifically, after full adjustment, compared with the first quartile, the HRs for cancer mortality were 0.84 (95% CI, 0.70–1.02) in the second quartile, 0.81 (95% CI, 0.68–0.97) in the third quartile, and 0.80 (95% CI, 0.68–0.95) in the fourth quartile (Table 2). The data about the association between serum calcium with other less common disease-specific mortality was not shown.

### The non-linear analysis of the association between serum calcium with mortality

**FIGURE 2:**
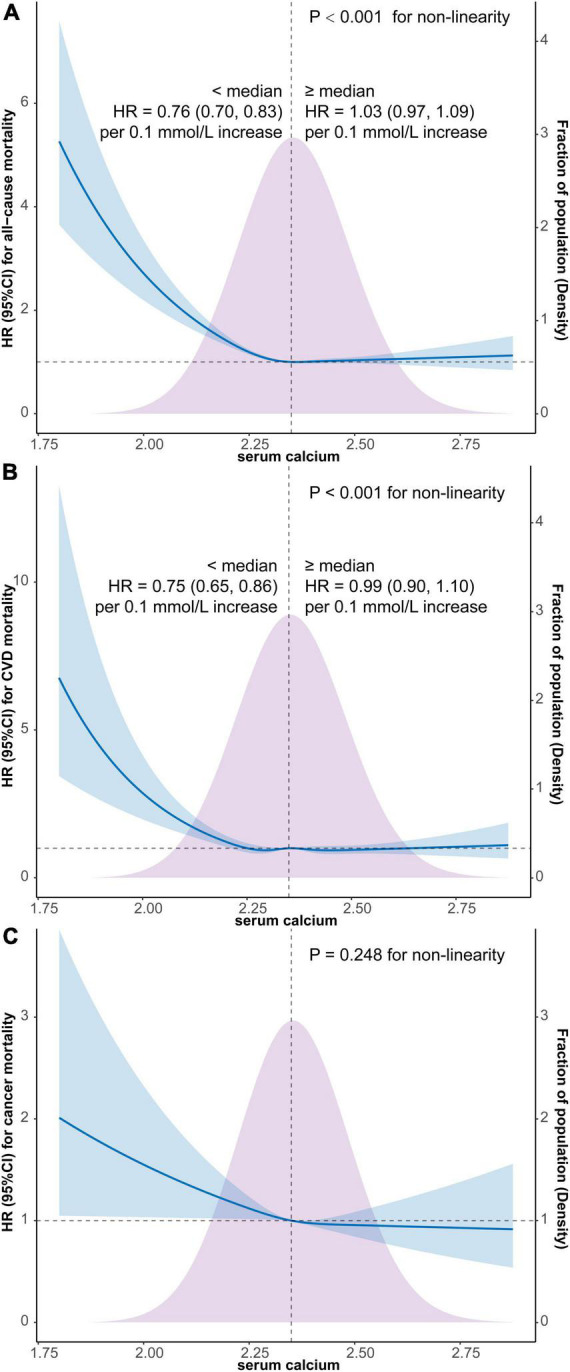
Survey-weighted multivariate Cox regression with restricted cubic spline analyses of the associations of continuous serum calcium level with all-cause mortality **(A)**, CVD mortality **(B)**, and cancer mortality **(C)** and segmented weighted Cox regression on both sides of the reference value. The models were all adjusted for sex, age, race, education, marital status, PIR, BMI, smoking, alcohol use, caffeine consumption, dietary calcium intake, HEI-2015, physical activity, comorbidity or history of hypertension, diabetes, CHD, stroke, COPD, cancer, and CKD. Solid blue lines are multivariable-adjusted HR estimations and the shaded areas are the corresponding 95% CIs. The violet-shaded area indicates the distribution of the population with different levels of calcium. HR, hazard ratio; CI, confidential interval; CVD, cardiovascular disease; BMI, body mass index; PIR, family income-to-poverty ratio; HEI, healthy eating index; COPD, chronic obstructive pulmonary disease; CHD, coronary heart disease; CKD, chronic kidney disease.

As depicted in Figure 2, the results of weighted multivariate Cox regression with restricted cubic spline (4 knots) found an L-shaped relationship between serum calcium and all-cause mortality (*P* < 0.001 for non-linearity), with the all-cause mortality reaching the reflection point approximately at the median position of serum calcium density distribution (Figure 2A). Additionally, the segmented weighted Cox proportional hazards model on both sides of the reference value (median of serum calcium) showed that below 2.35 mmol/L, the HR for all-cause mortality was 0.76 (95% CI, 0.70–0.83) per 0.1 mmol/L increments of serum calcium, while above 2.35 mmol the HR was 1.03 (95% CI, 0.97–1.09) per 0.1 mmol/L increments of serum calcium.

Similarly, an L-shaped relationship between serum calcium and CVD mortality was also detected by restricted cubic spline Cox regression with 5 knots (*P* < 0.001 for non-linearity), with the CVD mortality also reaching the reflection point approximately at the median position of serum calcium density distribution (Figure 2B). Furthermore, the segmented weighted Cox model showed that below 2.35 mmol/L, the HR for CVD mortality was 0.75 (95% CI, 0.65–0.86) per 0.1 mmol/L increments of serum calcium, while above 2.35 mmol the HR was 0.99 (95% CI, 0.90–1.10) per 0.1 mmol/L increments of serum calcium.

The weighted multivariate Cox regression with restricted cubic spline did not find a significant non-linear relationship between cancer mortality and serum calcium level (*P* = 0.248 for non-linearity) (Figure 2C).

### Subgroup analysis and interaction analysis

The associations of serum calcium levels in different subgroups stratified by dichotomic covariates with all-cause mortality were shown in Table 3. Thereinto, differences in all-cause mortality risk between subgroups indicated that the amount of dietary calcium intake and comorbidity of hypertension may have additional interaction with serum calcium in association with all-cause mortality.

**TABLE 3:** Subgroup analyses of the associations of serum calcium quartiles with all-cause mortality.

Subgroup		HR (95% CI) for all-cause mortality in serum calcium quartiles				
	No. of participants	Q1 [≤2.299]	Q2 [2.300, 2.349]	Q3 [2.350, 2.425]	Q4 [≥2.425]	*P* for interaction
Gender						0.859
Female	25,621	1 (ref)	0.77 (0.62, 0.97)	0.85 (0.68, 1.05)	0.87 (0.71, 1.06)	
Male	25,421	1 (ref)	0.82 (0.69, 0.99)	0.83 (0.69, 0.99)	0.87 (0.73, 1.03)	
Age						0.353
≤60	35,488	1 (ref)	0.89 (0.68, 1.15)	0.84 (0.64, 1.10)	0.87 (0.65, 1.15)	
>60	15,554	1 (ref)	0.75 (0.64, 0.89)	0.82 (0.71, 0.94)	0.80 (0.69, 0.94)	
Race						0.753
White	22,192	1 (ref)	0.83 (0.70, 0.97)	0.85 (0.72, 1.00)	0.90 (0.76, 1.06)	
Non-white	28,850	1 (ref)	0.73 (0.53, 0.99)	0.80 (0.62, 1.02)	0.76 (0.61, 0.95)	
Education						0.247
High school or below	23,850	1 (ref)	0.92 (0.74, 1.13)	0.87 (0.71, 1.05)	0.87 (0.71, 1.06)	
College or above	23,623	1 (ref)	0.73 (0.59, 0.90)	0.84 (0.69, 1.03)	0.90 (0.73, 1.11)	
Marital status						0.211
Married	24,912	1 (ref)	0.88 (0.72, 1.07)	0.93 (0.77, 1.12)	0.94 (0.79, 1.12)	
other	24,111	1 (ref)	0.72 (0.58, 0.90)	0.73 (0.59, 0.89)	0.78 (0.64, 0.95)	
PIR						0.054
<1.3	14,908	1 (ref)	0.96 (0.72, 1.27)	0.92 (0.71, 1.19)	0.81 (0.65, 1.02)	
≥1.3	31,662	1 (ref)	0.76 (0.64, 0.90)	0.80 (0.68, 0.96)	0.89 (0.75, 1.04)	
BMI						0.055
≤28	26,346	1 (ref)	0.71 (0.58, 0.87)	0.72 (0.60, 0.85)	0.75 (0.63, 0.89)	
>28	23,811	1 (ref)	0.91 (0.74, 1.12)	0.98 (0.80, 1.20)	1.03 (0.83, 1.27)	
Smoker						0.089
Non-smoker	26,256	1 (ref)	0.72 (0.58, 0.89)	0.76 (0.62, 0.94)	0.72 (0.59, 0.89)	
Smoker	22,009	1 (ref)	0.87 (0.72, 1.05)	0.91 (0.77, 1.07)	0.97 (0.82, 1.15)	
Current drinker						0.067
No	14,134	1 (ref)	0.95 (0.77, 1.18)	0.93 (0.76, 1.13)	1.03 (0.85, 1.26)	
Yes	29,628	1 (ref)	0.73 (0.60, 0.87)	0.79 (0.66, 0.94)	0.78 (0.65, 0.94)	
Caffeine consumption						0.429
≤P50	21,822	1 (ref)	0.87 (0.68, 1.11)	0.93 (0.74, 1.16)	0.89 (0.73, 1.09)	
>P50	21,657	1 (ref)	0.78 (0.65, 0.92)	0.80 (0.66, 0.95)	0.87 (0.73, 1.04)	
Dietary calcium intake (mg/day)						0.036
<800	22,341	1 (ref)	0.79 (0.65, 0.95)	0.94 (0.79, 1.13)	0.94 (0.79, 1.11)	
≥800	21,138	1 (ref)	0.84 (0.68, 1.05)	0.73 (0.60, 0.90)	0.81 (0.65, 1.02)	
HEI2015						0.126
≤50	24,417	1 (ref)	0.87 (0.73, 1.03)	0.86 (0.71, 1.05)	0.81 (0.67, 0.98)	
>50	23,598	1 (ref)	0.75 (0.60, 0.92)	0.82 (0.69, 0.97)	0.93 (0.77, 1.12)	
Physical activity						0.936
Inactive	13,112	1 (ref)	0.82 (0.68, 1.00)	0.81 (0.67, 0.99)	0.87 (0.72, 1.07)	
Active	23,678	1 (ref)	0.81 (0.65, 1.01)	0.85 (0.70, 1.03)	0.88 (0.73, 1.06)	
Hypertension						0.025
No	30,064	1 (ref)	0.71 (0.56, 0.91)	0.77 (0.61, 0.98)	0.70 (0.55, 0.90)	
Yes	20,978	1 (ref)	0.88 (0.74, 1.05)	0.89 (0.76, 1.05)	0.99 (0.83, 1.18)	
Diabetes						0.69
No	42,516	1 (ref)	0.78 (0.66, 0.92)	0.81 (0.70, 0.95)	0.86 (0.73, 1.01)	
Yes	8,522	1 (ref)	0.95 (0.71, 1.29)	1.02 (0.78, 1.33)	0.95 (0.72, 1.26)	
CHD						0.456
No	45,284	1 (ref)	0.82 (0.71, 0.96)	0.82 (0.70, 0.96)	0.86 (0.74, 1.01)	
Yes	2,043	1 (ref)	0.67 (0.46, 0.99)	0.94 (0.66, 1.32)	0.88 (0.60, 1.29)	
Stroke						0.29
No	45,645	1 (ref)	0.81 (0.70, 0.93)	0.85 (0.74, 0.99)	0.87 (0.75, 1.01)	
Yes	1,837	1 (ref)	0.95 (0.58, 1.55)	0.84 (0.51, 1.38)	1.12 (0.67, 1.86)	
COPD						0.521
No	45,561	1 (ref)	0.80 (0.69, 0.93)	0.84 (0.72, 0.97)	0.85 (0.73, 0.99)	
Yes	2,011	1 (ref)	0.84 (0.54, 1.30)	0.81 (0.50, 1.32)	0.98 (0.62, 1.54)	
Cancer						0.686
No	43,089	1 (ref)	0.80 (0.68, 0.95)	0.82 (0.71, 0.96)	0.85 (0.72, 1.01)	
Yes	4,404	1 (ref)	0.86 (0.65, 1.15)	0.94 (0.71, 1.25)	0.98 (0.75, 1.29)	
CKD						0.266
No	41,277	1 (ref)	0.77 (0.64, 0.92)	0.84 (0.71, 1.00)	0.82 (0.69, 0.98)	
Yes	9,217	1 (ref)	0.88 (0.70, 1.11)	0.84 (0.69, 1.03)	0.96 (0.77, 1.20)	

CI, confidence interval; BMI, body mass index; PIR, family income-to-poverty ratio; P50, 50th percentile; HEI, healthy eating index; COPD, chronic obstructive pulmonary disease; CHD, coronary heart disease; CKD, chronic kidney disease.

Similarly, differences in CVD mortality risk between subgroups indicated that categorical age, HEI-2015, and comorbidity of hypertension may have additional interactions with serum calcium in association with CVD mortality (Table 4).

**TABLE 4:** Subgroup analyses of the associations of serum calcium quartiles with CVD mortality.

Subgroup		HR (95% CI) for CVD mortality in serum calcium quartiles				
	No. of participants	Q1 [≤2.299]	Q2 [2.300, 2.349]	Q3 [2.350, 2.425]	Q4 [≥2.425]	*P* for interaction
Gender						0.099
Female	25,621	1 (ref)	0.79 (0.52, 1.21)	0.65 (0.45, 0.93)	0.72 (0.50, 1.04)	
Male	25,421	1 (ref)	0.80 (0.61, 1.05)	1.00 (0.76, 1.30)	0.87 (0.67, 1.13)	
Age						0.004
≤60	35,488	1 (ref)	1.07 (0.54, 2.11)	1.61 (0.90, 2.88)	0.92 (0.44, 1.96)	
>60	15,554	1 (ref)	0.75 (0.57, 1.00)	0.65 (0.51, 0.84)	0.72 (0.57, 0.91)	
Race						0.343
White	22,192	1 (ref)	0.76 (0.58, 1.01)	0.89 (0.70, 1.12)	0.81 (0.62, 1.05)	
Non-white	28,850	1 (ref)	0.98 (0.60, 1.60)	0.68 (0.44, 1.05)	0.92 (0.56, 1.49)	
Education						0.179
High school or below	23,850	1 (ref)	0.99 (0.69, 1.41)	0.82 (0.59, 1.14)	0.80 (0.58, 1.10)	
College or above	23,623	1 (ref)	0.65 (0.45, 0.93)	0.96 (0.68, 1.36)	0.90 (0.63, 1.30)	
Marital status						0.845
Married	24,912	1 (ref)	0.81 (0.58, 1.12)	0.91 (0.68, 1.23)	0.84 (0.63, 1.13)	
other	24,111	1 (ref)	0.79 (0.53, 1.16)	0.77 (0.56, 1.06)	0.81 (0.58, 1.14)	
PIR						0.37
<1.3	14,908	1 (ref)	1.22 (0.74, 2.03)	0.90 (0.57, 1.43)	1.00 (0.67, 1.49)	
≥1.3	31,662	1 (ref)	0.71 (0.54, 0.93)	0.82 (0.64, 1.06)	0.78 (0.60, 1.02)	
BMI						0.554
≤28	26,346	1 (ref)	0.81 (0.56, 1.17)	0.83 (0.58, 1.19)	0.89 (0.64, 1.22)	
>28	23,811	1 (ref)	0.77 (0.55, 1.09)	0.83 (0.60, 1.16)	0.69 (0.50, 0.97)	
Smoker						0.303
Non-smoker	26,256	1 (ref)	0.63 (0.43, 0.94)	0.82 (0.57, 1.17)	0.67 (0.47, 0.95)	
Smoker	22,009	1 (ref)	0.94 (0.69, 1.28)	0.89 (0.68, 1.18)	0.92 (0.67, 1.26)	
Current drinker						0.611
No	14,134	1 (ref)	0.87 (0.58, 1.32)	0.86 (0.60, 1.23)	0.96 (0.69, 1.33)	
Yes	29,628	1 (ref)	0.76 (0.55, 1.04)	0.83 (0.62, 1.13)	0.72 (0.54, 0.98)	
Caffeine consumption						0.856
≤P50	21,822	1 (ref)	0.73 (0.51, 1.05)	0.85 (0.62, 1.18)	0.79 (0.56, 1.11)	
>P50	21,657	1 (ref)	0.85 (0.61, 1.18)	0.86 (0.64, 1.16)	0.88 (0.66, 1.16)	
Dietary calcium intake (mg/day)						0.235
<800	22,341	1 (ref)	0.74 (0.54, 1.01)	0.76 (0.57, 1.02)	0.69 (0.52, 0.90)	
≥800	21,138	1 (ref)	0.95 (0.65, 1.40)	1.03 (0.73, 1.46)	1.07 (0.76, 1.50)	
HEI2015						0.032
≤50	24,417	1 (ref)	1.04 (0.71, 1.53)	1.05 (0.75, 1.46)	0.81 (0.57, 1.13)	
>50	23,598	1 (ref)	0.65 (0.47, 0.91)	0.73 (0.55, 0.96)	0.85 (0.64, 1.13)	
Physical activity						0.95
Inactive	13,112	1 (ref)	0.87 (0.62, 1.22)	0.91 (0.65, 1.28)	0.82 (0.61, 1.11)	
Active	23,678	1 (ref)	0.78 (0.54, 1.11)	0.86 (0.65, 1.15)	0.86 (0.62, 1.19)	
Hypertension						0.006
No	30,064	1 (ref)	0.63 (0.39, 1.02)	0.94 (0.64, 1.39)	0.51 (0.32, 0.82)	
Yes	20,978	1 (ref)	0.88 (0.66, 1.19)	0.84 (0.66, 1.08)	0.97 (0.74, 1.27)	
Diabetes						0.732
No	42,516	1 (ref)	0.75 (0.55, 1.03)	0.82 (0.64, 1.06)	0.81 (0.62, 1.06)	
Yes	8,522	1 (ref)	1.03 (0.67, 1.59)	1.13 (0.76, 1.68)	0.89 (0.56, 1.42)	
CHD						0.843
No	45,284	1 (ref)	0.84 (0.62, 1.14)	0.88 (0.69, 1.12)	0.85 (0.64, 1.12)	
Yes	2,043	1 (ref)	0.66 (0.39, 1.11)	0.71 (0.42, 1.20)	0.67 (0.39, 1.16)	
Stroke						0.83
No	45,645	1 (ref)	0.80 (0.62, 1.03)	0.87 (0.68, 1.10)	0.81 (0.64, 1.04)	
Yes	1,837	1 (ref)	1.07 (0.43, 2.69)	0.86 (0.25, 2.94)	1.30 (0.47, 3.60)	
COPD						0.583
No	45,561	1 (ref)	0.76 (0.59, 0.98)	0.85 (0.68, 1.06)	0.79 (0.63, 1.00)	
Yes	2,011	1 (ref)	1.51 (0.68, 3.36)	0.99 (0.42, 2.35)	1.26 (0.50, 3.20)	
Cancer						0.132
No	43,089	1 (ref)	0.78 (0.59, 1.03)	0.82 (0.64, 1.05)	0.74 (0.57, 0.96)	
Yes	4,404	1 (ref)	0.98 (0.54, 1.77)	1.16 (0.69, 1.94)	1.37 (0.82, 2.29)	
CKD						0.156
No	41,277	1 (ref)	0.73 (0.53, 1.01)	0.97 (0.73, 1.30)	0.81 (0.60, 1.10)	
Yes	9,217	1 (ref)	0.88 (0.61, 1.27)	0.69 (0.52, 0.94)	0.83 (0.59, 1.16)	

CI, confidence interval; BMI, body mass index; PIR, family income-to-poverty ratio; P50, 50th percentile; HEI, healthy eating index; COPD, chronic obstructive pulmonary disease; CHD, coronary heart disease; CKD, chronic kidney disease.

### Sensitivity analyses

Repeating the main analyses showed that, after excluding participants with potential outlier serum calcium, the differences in all-cause mortality, CVD mortality, and cancer mortality in serum calcium quartiles kept robust (Supplementary Table 3). Furthermore, the non-linear associations of serum calcium with all-cause and CVD mortality did not show any substantial changes after excluding these participants (Supplementary Figure 1).

Similarly, after excluding participants who died within 2 years, the differences in all-cause mortality, CVD mortality, and cancer mortality in serum calcium quartiles also kept robust (Supplementary Table 4); the non-linear associations of serum calcium with all-cause and CVD mortality almost kept the same after excluding these participants (Supplementary Figure 2).

When participants with missing data of covariates were excluded, the associations of categorical serum calcium with all-cause mortality kept robust, while the associations with CVD mortality were generally stable but attenuated in the fully adjusted model; however, the associations with cancer mortality were all weakened in adjusted models from point of statistics (Supplementary Table 5). Likewise, the non-linear associations of serum calcium with all-cause and CVD mortality also kept robust after excluding these participants (Supplementary Figure 3).

## Discussion

In this large prospective cohort study of the US general adult population, survey-weighted Cox regression models with multiple adjustments of covariates showed the constant higher risk of all-cause mortality and CVD mortality in the first quantile of serum calcium. Furthermore, the non-linearity analyses demonstrated the L-shaped associations of continuous serum calcium with all-cause mortality and CVD mortality, but not cancer mortality. The concentration of total serum calcium with the lowest risk of all-cause and CVD mortality was approximately the median of the serum calcium of the population (2.35 mmol/L). The risk of all-cause and CVD mortality increases dramatically when serum calcium was below 2.35 mmol/L, while the risk of all-cause and CVD mortality kept relatively flat when serum calcium was above 2.35 mmol/L, with extensive potential covariates adjusted.

In this nationally representative non-pregnant adult cohort, the serum calcium was distributed in a relatively narrow range, with more than 95% values located in the reference range, 2.20–2.60 mmol/L (8.8–10.4 mg/dL) (3). The tightly controlled calcium hemostasis reflected the crucial role of calcium in maintaining principal cellular functions and variances in serum calcium may have significant impacts on organic or systemic physiological function. It should be noted that the serum calcium level may have some subtle fluctuations due to sex, age, or race, which was shown in our results and previous research (24, 25), as well as some lifestyle factors (26) and clinical situations (27).

The association between low serum calcium and an increased risk of all-cause mortality has been investigated in various hospital settings. As reported, low serum calcium was an independent predictor of all-cause mortality in myocardial infarction patients (28), in patients with chronic heart failure (5), in patients with coronary heart disease (6), in patients following acute pulmonary thromboembolism (29), in neonatal sepsis patients (30), in elderly patients with sepsis (31), and in critically ill patients with acute kidney injury (32). These findings indicated the extensive role of extracellular calcium and the potentially detrimental effect of low serum calcium. The evidence from large-scale general populations was rare, but two approximate studies, one from the Copenhagen General Population Study (*n* = 106,768) (33) and the other from a US veteran cohort (*n* = 1,967,622) (25) also reported the associations of low serum calcium with all-cause mortality.

Concerning the risk of CVD mortality, Yarmohammadi, H. reported that low serum calcium was an independent risk factor of sudden cardiac arrest in the community (34), which was probably due to the QT prolongation and mechanical cardiac dysfunction implicated by lower calcium (35). Van Hemelrijck, M. reported the increased risk of overall CVD death for those with low serum calcium, especially the increased ischemic heart mortality in men from the third National Health and Nutrition Examination Survey (NHANES III) (36). For cerebrovascular events, it was reported that lower serum calcium was associated with the increased risk of ischemic stroke (37), and among patients with intracerebral hemorrhage, those with hypocalcemia tended to have larger hematoma volume (38, 39), which may be related to the coagulopathy mediated by hypocalcemia (38).

In our study, the differences in cancer mortality between serum calcium level quartiles did not keep robust, which was possible due to the heterogeneity of cancer. It was reported that subjects with higher serum calcium tended to have a reduced risk of cancer incidence from the Tromsø Study (40) and similar research showed the inverse association between total serum calcium and breast cancer from the Swedish Apolipoprotein Mortality Risk (AMORIS) Study (41). However, different results were also observed in other studies, as a higher risk for ovarian cancer mortality with increments in total serum calcium was reported from the Third National Health and Nutrition Survey (NHANES III) (42) and the positive relation between serum calcium and incidence of esophageal cancer and colorectal cancer was found from the Swedish AMORIS study (43). So, further studies focusing on certain types of cancer are still needed to obtain definite conclusions.

In the present study, the risk of all-cause mortality and CVD mortality did not show a significant increment when serum calcium was above 2.35 mmol/L, which seems not consistent with other studies (44, 45). In previous studies, Larsson, T. E Wang. reported higher serum calcium is associated with a higher risk of total and non-cardiovascular mortality from an adult men cohort (*n* = 2,322) in Uppsala, Sweden (46). The inconsistent findings could be partially due to the differences in gender distribution and population sources. In another research, Lu, J. L. reported a U-shaped association between serum calcium and all-cause mortality, in which high serum calcium was also associated with a high risk of all-cause mortality. In our models, we included more covariates, such as physical activity, which could impact both serum calcium (47) and mortality, which may partially explain the disparity. Similarly, Lutsey, P. L. reported a greater risk of heart failure incidence with higher concentrations of calcium from the Atherosclerosis Risk in Communities (ARIC) Study (48), but it should be noted that this effect was most significant when serum calcium higher than 10.2 mg/dL (2.55 mmol/L), which constituted a relatively low proportion in our study. To sum up, although the potentially detrimental effect of high serum calcium was not detected from this specific cohort with such population component and serum calcium distribution, extreme caution should be exercised when trying to generalize the results.

In our present study, we chose the median, 2.35 mmol/L, as the reference value, which was shown to be close to the optimal point with the lowest risk of all-cause mortality and disease-specific mortality. However, the optimal serum calcium level in different populations or different clinical situations should be re-determined. The subgroup analysis indicated that the all-cause mortality and CVD mortality risk showed different relations with serum calcium quartiles in different subgroup populations; for instance, the association of lower serum calcium with higher all-cause mortality and CVD mortality was more prominent in adults without hypertension. Concerning the sensitivity analysis of our results, the non-linear relationship of serum calcium with all-cause mortality and CVD mortality kept rather stable even if excluding participants with potential outliers, with missing data of covariates, or dying within 2 years. For the categorical serum calcium quartiles, the association of serum calcium with all-cause mortality kept robust even if excluding participants with potential outliers, with missing data of covariates, or dying within 2 years; while the association of serum calcium quartiles with CVD mortality kept robust after excluding participants with potential outliers or dying within 2 years but was weakened after excluding participants with missing data of covariates, indicating the association of serum calcium with CVD mortality may need further confirmation in more complete datasets.

There are some strengths in our present study. Firstly, our study finally included a relatively large sample size of participants (>50,000) with an average of 9.7 years follow-up which is rare in studies focusing on the association of serum calcium level with mortality. Secondly, because the participants included in NHANES were nationally representative, our results should be universal to the US adult population. Thirdly, extensive covariates were taken into consideration in our models with linear and non-linear statistical analysis, so our conclusion should be robust and rational.

However, several limitations of our study must be acknowledged. Firstly, all participants were sampled from US non-institutional civilians, which may limit the applicability of our results to other populations. Secondly, our results reflected the association of serum calcium level with mortality drawn from the general population, but not the causal relationship, so it cannot be used to guide clinical management for specific patients under specific clinical situations. Thirdly, the serum calcium level was based on a single measurement, which might be fluctuated by some transit factors and may not accurately reflect the long-term status, so repeated measurement should be advocated in future investigations. Finally, residual or unmeasured confounding cannot be entirely excluded despite every effort made to adjust the major confounding factors.

## Conclusion

From a nationally representative sample of US adults, we found an L-shaped association of serum calcium levels with all-cause and CVD mortality. When serum calcium was lower than the median, the risk of all-cause and CVD mortality increases dramatically; while the risk of all-cause and CVD mortality increased slightly when serum calcium was above the median. Our results indicated a potential beneficial effect of maintaining the appropriate serum calcium level in decreasing the risk of all-cause and CVD mortality.

## Data availability statement

The original contributions presented in this study are included in the article/Supplementary material, further inquiries can be directed to the corresponding author.

## Ethics statement

The protocols of NHANES were approved by NCHS Ethics Review Board, CDC (https://www.cdc.gov/nchs/nhanes/irba98.htm), and written informed consent was obtained from all participants.

## Author contributions

XH and JH analyzed and interpreted the data. XH, ZL, and ZZ prepared the manuscript. QG and EW edited the manuscript. ZS conceived the study design and responsible for the overall content. All authors contributed to the article and approved the submitted version.

## Footnotes

1
http://www.r-project.org

2
www.empowerstats.net

## Supplementary material

Supplementary Figure 1 Survey-weighted multivariate Cox regression with restricted cubic spline analyses of the associations of continuous serum calcium level with all-cause mortality **(A)**, CVD mortality **(B)**, and cancer mortality **(C)**, and segmented weighted Cox regression on both sides of the reference value after excluding potential outliers. The models were all fully adjusted for the mentioned covariates. Solid blue lines are multivariable-adjusted HR estimations and the shaded areas are the corresponding 95% CIs. The violet-shaded area indicates the distribution of the population with different levels of calcium. HR, hazard ratio; CI, confidential interval; CVD, cardiovascular disease.

Supplementary Figure 2 Survey-weighted multivariate Cox regression with restricted cubic spline analyses of the associations of continuous serum calcium level with all-cause mortality **(A)**, CVD mortality **(B)**, and cancer mortality **(C)**, and segmented weighted Cox regression on both sides of the reference value after excluding participants who died within 2 years. The models were all fully adjusted for the mentioned covariates. Solid blue lines are multivariable-adjusted HR estimations and the shaded areas are the corresponding 95% CIs. The violet-shaded area indicates the distribution of the population with different levels of calcium. HR, hazard ratio; CI, confidential interval; CVD, cardiovascular disease.

Supplementary Figure 3 Survey-weighted multivariate Cox regression with restricted cubic spline analyses of the associations of continuous serum calcium level all-cause mortality **(A)**, CVD mortality **(B)**, and cancer mortality **(C)**, and segmented weighted Cox regression on both sides of the reference value after the missing data of covariates imputed by the multiple imputation method. The models were all fully adjusted for the mentioned covariates. Solid blue lines are multivariable-adjusted HR estimations and the shaded areas are the corresponding 95% CIs. The violet-shaded area indicates the distribution of the population with different levels of calcium. HR, hazard ratio; CI, confidential interval; CVD, cardiovascular disease.
