# Supplementary figures and images for: L-shaped association of serum calcium with all-cause and CVD mortality in the US adults: A population-based prospective cohort study
Source: Front Nutr. 2023 Jan 5;9:1097488. doi: 10.3389/fnut.2022.1097488 (PMC9849810; doi:10.3389/fnut.2022.1097488)

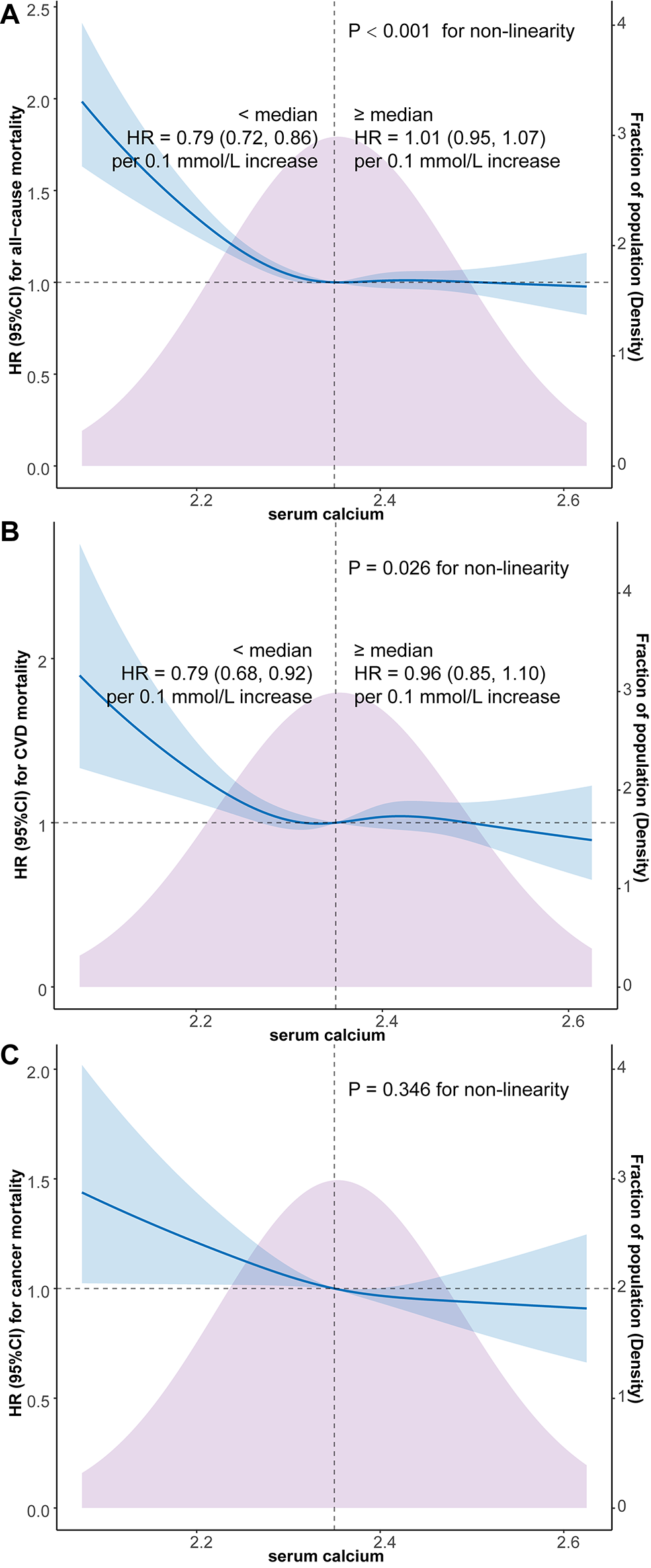

Supplement: Supplementary Figure 1 — Survey-weighted multivariate Cox regression with restricted cubic spline analyses of the associations of continuous serum calcium level with all-cause mortality (A), CVD mortality (B), and cancer mortality (C), and segmented weighted Cox regression on both sides of the reference value after excluding potential outliers. The models were all fully adjusted for the mentioned covariates. Solid blue lines are multivariable-adjusted HR estimations and the shaded areas are the corresponding 95% CIs. The violet-shaded area indicates the distribution of the population with different levels of calcium. HR, hazard ratio; CI, confidential interval; CVD, cardiovascular disease. [file Image_1.TIF]

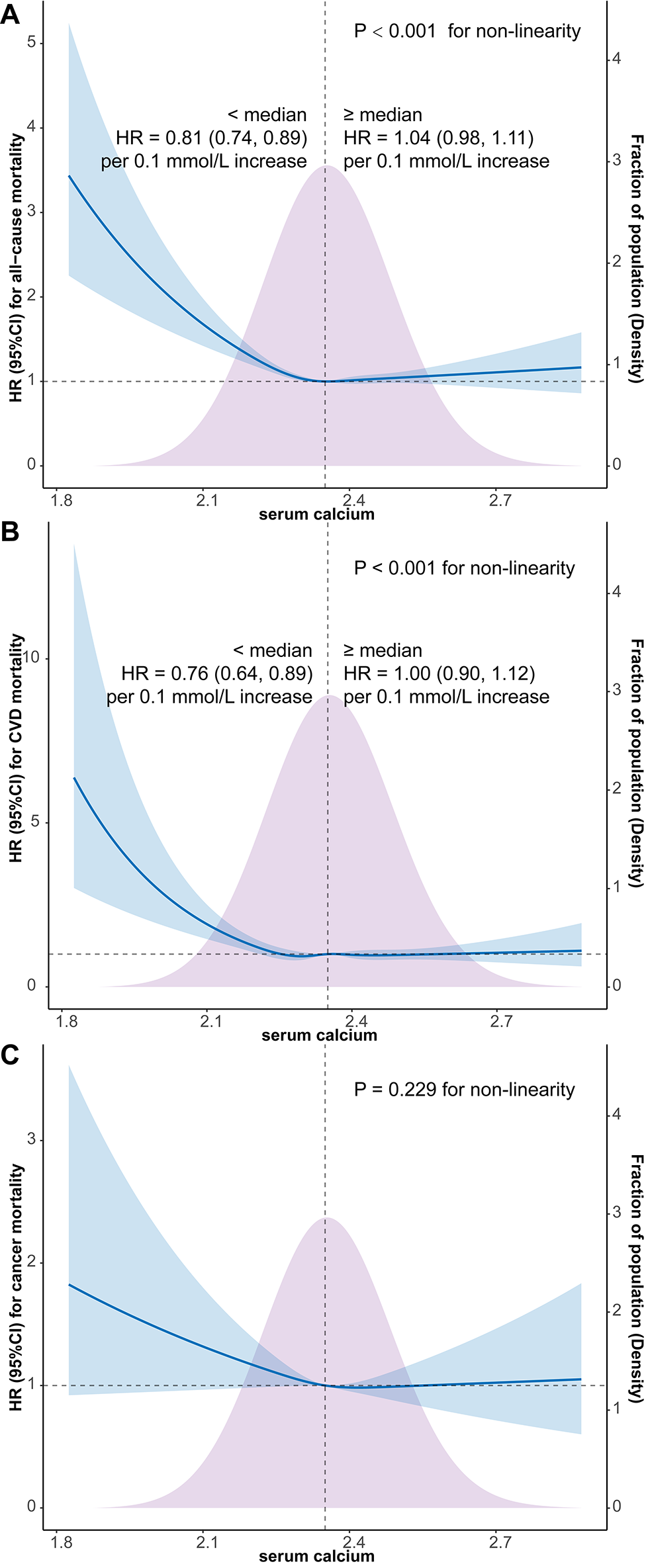

Supplement: Supplementary Figure 2 — Survey-weighted multivariate Cox regression with restricted cubic spline analyses of the associations of continuous serum calcium level with all-cause mortality (A), CVD mortality (B), and cancer mortality (C), and segmented weighted Cox regression on both sides of the reference value after excluding participants who died within 2 years. The models were all fully adjusted for the mentioned covariates. Solid blue lines are multivariable-adjusted HR estimations and the shaded areas are the corresponding 95% CIs. The violet-shaded area indicates the distribution of the population with different levels of calcium. HR, hazard ratio; CI, confidential interval; CVD, cardiovascular disease. [file Image_2.TIF]

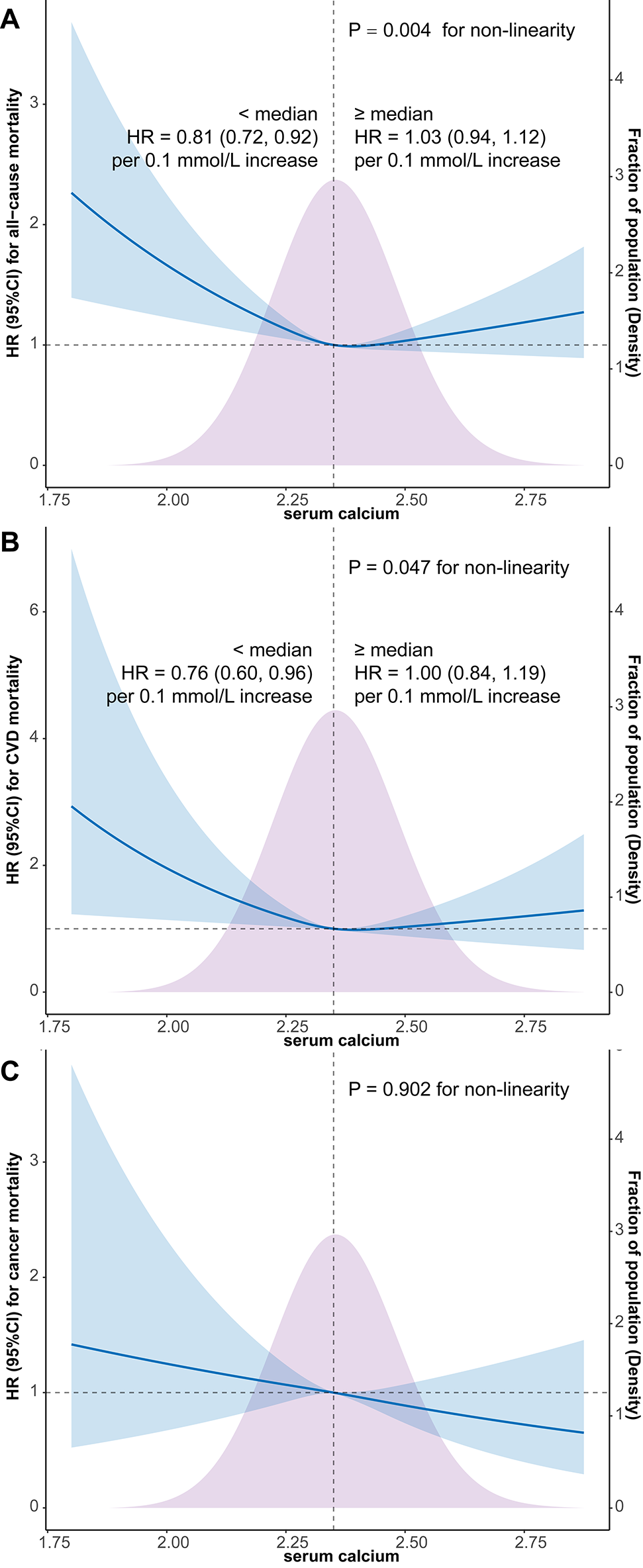

Supplement: Supplementary Figure 3 — Survey-weighted multivariate Cox regression with restricted cubic spline analyses of the associations of continuous serum calcium level all-cause mortality (A), CVD mortality (B), and cancer mortality (C), and segmented weighted Cox regression on both sides of the reference value after the missing data of covariates imputed by the multiple imputation method. The models were all fully adjusted for the mentioned covariates. Solid blue lines are multivariable-adjusted HR estimations and the shaded areas are the corresponding 95% CIs. The violet-shaded area indicates the distribution of the population with different levels of calcium. HR, hazard ratio; CI, confidential interval; CVD, cardiovascular disease. [file Image_3.TIF]
